# Short-Term Exercise Progression of Cardiovascular Patients throughout Cardiac Rehabilitation: An Observational Study

**DOI:** 10.3390/jcm9103160

**Published:** 2020-09-29

**Authors:** Hélène De Cannière, Christophe J. P. Smeets, Melanie Schoutteten, Carolina Varon, John F. Morales Tellez, Chris Van Hoof, Sabine Van Huffel, Willemijn Groenendaal, Pieter Vandervoort

**Affiliations:** 1 Mobile Health Unit, Faculty of Medicine and Life Sciences, Hasselt University, 3500 Hasselt, Belgium; Christophe.smeets@uhasselt.be (C.J.P.S.); Melanie.schoutteten@uhasselt.be (M.S.); pieter.vandervoort@zol.be (P.V.); 2 Future Health Department, Ziekenhuis Oost-Limburg, 3600 Genk, Belgium; 3 Imec the Netherlands/Holst Centre, 5656AE Eindhoven, The Netherlands; Willemijn.groenendaal@imec.nl; 4 KU Leuven, Department of Electrical Engineering (ESAT), STADIUS Center for Dynamical Systems, Signal Processing and Data Analytics, 3001 Leuven, Belgium; carolina.varon@esat.kuleuven.be (C.V.); johnfredy.moralestellez@esat.kuleuven.be (J.F.M.T.); chris.vanhoof@imec.be (C.V.H.); sabine.vanhuffel@esat.kuleuven.be (S.V.H.); 5 TU Delft, Department of Microelectronics, Circuits and Systems (CAS), 2600AA Delft, The Netherlands; 6 Imec Vzw Belgium, 3001 Leuven, Belgium; 7 Department of Cardiology, Ziekenhuis Oost-Limburg, 3600 Genk, Belgium

**Keywords:** cardiovascular disease, cardiac rehabilitation, functional capacity, 6MWT

## Abstract

Cardiac rehabilitation (CR) is a highly recommended secondary prevention measure for patients with diagnosed cardiovascular disease. Unfortunately, participation rates are low due to enrollment and adherence issues. As such, new CR delivery strategies are of interest, as to improve overall CR delivery. The goal of the study was to obtain a better understanding of the short-term progression of functional capacity throughout multidisciplinary CR, measured as the change in walking distance between baseline six-minute walking test (6MWT) and four consecutive follow-up tests. One-hundred-and-twenty-nine patients diagnosed with cardiovascular disease participated in the study, of which 89 patients who completed the whole study protocol were included in the statistical analysis. A one-way repeated measures ANOVA was conducted to determine whether there was a significant change in mean 6MWT distance (6MWD) throughout CR. A three-way-mixed ANOVA was performed to determine the influence of categorical variables on the progression in 6MWD between groups. Significant differences in mean 6MWD between consecutive measurements were observed. Two subgroups were identified based on the change in distance between baseline and end-of-study. Patients who increased most showed a linear progression. In the other group progression leveled off halfway through rehabilitation. Moreover, the improvement during the initial phase of CR seemed to be indicative for overall progression. The current study adds to the understanding of the short-term progression in exercise capacity of patients diagnosed with cardiovascular disease throughout a CR program. The results are not only of interest for CR in general, but could be particularly relevant in the setting of home-based CR.

## 1. Introduction

Cardiac rehabilitation (CR) is an evidence-based intervention that uses a multidisciplinary approach to improve secondary prevention outcomes in cardiovascular patients [1,2,3,4,5]. The clinical effectiveness of conventional center-based CR is well established and the beneficial effects of CR on mortality and hospital readmissions have been extensively discussed [6,7,8,9,10,11]. Although a large and increasing number of cardiovascular patients need CR, many will not participate due to issues such as limited referral, enrolment problems or suboptimal completion rates [12]. Participation rates are especially low for women, elderly patients, and socially disadvantaged groups [1,13]. New CR delivery strategies are needed to improve enrollment, adherence, and completion in eligible patients. However, the heterogeneity in CR programs implemented across Europe makes it difficult to properly study and interpret the effects of the intervention. Moreover, to be able to accurately measure quality of care improvement, a CR standardization process is needed. The European Association of Preventive Cardiology (EAPC) accreditation program focusses on developing minimum standards for the evaluation of quality of a CR program, including characterization of eligible patients, ideal timing of CR and defining necessary components of CR [14]. These standardization procedures are also wanted within home-based CR. A potential new approach to overcome some of the current enrollment barriers is by moving CR to an in-home setting [14,15,16]. Several studies and meta-analyses have shown that home-based CR and center-based CR have similar effects on clinical primary outcomes, including total mortality, exercise capacity, and health-related quality of life. However, these analyses also report the heterogeneity of CR programs among studies as a major limitation, further emphasizing the need for standardization procedures [17,18,19]. Furthermore, parameters of care, defined by standardization, can contribute to the personalization of treatment strategies, optimizing the patients’ needs and preferences. In addition, wearable telemonitoring solutions support patients in following personalized treatment recommendations (i.e., exercise training, diet, medication…), both in home- and center-based CR programs [15].

Although the majority of studies on home- and center-based CR programs report data on changes in exercise capacity measured at baseline and on completion of the intervention limited information is available on the short-term progression in exercise capacity throughout the CR [20,21,22].

The six-minute walking test (6MWT) is one of the outcome measures used to report progression [23]. The 6MWT is often used as a testing modality in clinical practice due to its simplicity, safety, low-cost and ease-to-administer [23,24,25]. In addition, the 6MWT is reflective for activities of daily living and can thus be easily translated to a standardized activity in an in-home environment. Consequently, the 6MWT is not only suitable for studying short-term changes in exercise capacity, but it could also be used to report changes in exercise capacity at home [26,27]. However, the 6MWT is characterized by some limitations. Compared to the cardiopulmonary exercise test (CPET), which provides a global assessment of functional capacity, it is impossible to determine the causes or mechanisms of exercise limitation or dyspnea on exertion. However, despite these differences, good correlations have been shown between both tests [25,28].

In this study, we hypothesized that monitoring progression in detail during the course of a standardized rehabilitation program could provide more insight into the actual improvement in exercise capacity. To our knowledge, the present research is the first to study the effects of exercise training at short-time intervals in the course of a standardized CR program. Future studies could potentially use this information to optimize short-term guidance and treatment strategies, namely in home-based CR programs.

## 2. Materials and Methods

### 2.1. Study Population

One hundred and twenty-nine patients diagnosed with cardiovascular disease, participating in a structured ambulatory multidisciplinary CR program in a single tertiary care center (Ziekenhuis Oost-Limburg, Genk, Belgium), were included. Criteria for eligibility were being 18-years-old or older, providing informed consent, HF with reduced ejection fraction (EF), HF with preserved ejection fraction and patients referred to CR after myocardial infarction, coronary artery bypass grafting, percutaneous intervention or other, with a left ventricular EF less than or equal to 55%, measured during the baseline echocardiography measurement. The diagnosis (i.e., HF) were considered as according to the electronic patient records and as described by the patient’s physician. A cut-off value of 55% in terms of EF was chosen based on the values that are reported by Lang et al. Values below 55% are considered to be abnormal. This experience-based partition value allows to achieve meaningful clinical categorization of the severity of abnormality [29]. Exclusion criteria were orthopedic or neurological limitations. The study complied with the Declaration of Helsinki (Fortaleza, Brazil, 2013) and the local institutional ethical committee (CME ZOL Genk) approved the study protocol. On average, one out of ten eligible patients declined participation. Written informed consent was obtained from all participating subjects.

### 2.2. Standard of Care Multidisciplinary CR Program

After a cardiovascular-related hospitalization or consultation, patients were referred to the multidisciplinary CR program, according to standard of care in our center. Baseline cardiopulmonary exercise testing (CPET) on a stationary bicycle and echocardiography measurements (Philips Medical Systems, IE33, Andover, MA, USA) were performed prior to program enrollment. For CPET, a ramp protocol starting at 50 W and increasing 30 W every 2 min was used. Patients on beta-blocker therapy were asked to discontinue beta-blocker treatment prior to testing in order for their heart rate to be able to reach the age-predicted value. The modified Simpson’s biplane method was used to calculate EF. Moreover, clinical assessment was undertaken during CPET measurements at the beginning and end of the program. Enrolled patients had to follow 45-sessions of supervised ambulatory rehabilitation at a frequency of three one-hour-sessions a week. The training protocol consisted of both aerobic and resistive exercises. Seventy percent to 85% of the maximal heart rate (HR) was chosen as target HR during aerobic training. Aerobic training consisted of in total 30–40 min of aerobic exercise on bicycle, handbike, treadmill and/or stepper. Resistive training, added halfway into the rehabilitation program, was performed at 50–80% of one repetition maximum. Resistive training consisted of three times fifteen repetitions on both the leg and arm press. The supervising physiotherapist increased training intensity based on individual performance every two weeks. Additionally, when deemed necessary or upon patient request, dietary sessions, psychological support, and social consultations were included in the multidisciplinary program. A CPET was repeated and at the end of the rehabilitation program, together with an echocardiographic assessment. Adherence to the rehabilitation program was evaluated by measuring attendance at the exercise sessions.

### 2.3. Baseline Characteristics and Experimental Study Protocol

Demographic and clinical data (baseline CPET, New York Heart Association (NYHA)-class, medical therapy, baseline echocardiography, reason for referral, and medical history) were collected from the electronic medical record. Health-related quality of life (HRQoL) assessments were carried out using the Short Form-36 (SF-36) questionnaire and the Minnesota Living with Heart Failure Questionnaire (MLHFQ) at baseline and end-of-study, both validated in CR patients. A 6MWT was taken at baseline (start of rehabilitation program) and was repeated four times (every three weeks), for five times in total. The 6MWT was performed according to a standardized protocol [24]. The test was done in a 45-m-corridor and standardized encouraging sentences were repeated by the researcher every minute throughout the test. The distance traveled after completing the 6MWT was determined. In response to exercise training, a change in walking distance of 30–50 m is considered clinically relevant [24]. Both the patient’s self-assessment of wellbeing on a scale of one to ten and the NYHA classification were determined before. Hemodynamic parameters, including HR, SpO_2_ and blood pressure were assessed before and after the test. The Borg score of perceived exertion for dyspnea and fatigue was also recorded before and upon completion of the test. The baseline characteristics between patients who completed all five 6MWTs and patients who failed to finish the study protocol were compared.

### 2.4. Study-Endpoints

This study focused on the progression of functional capacity throughout a standardized rehabilitation program. The functional improvement was measured as the change in distance between baseline 6MWT and every consecutive follow-up test.

### 2.5. Statistics

Continuous variables were expressed as mean (standard deviation) if normally distributed or as median (interquartile range) if non-normally distributed and dichotomous data were expressed as n (%). Normality was assessed by the Shapiro-Wilk statistic. A one-way repeated measures ANOVA was conducted to determine whether there were statistically significant differences in mean 6MWT distance (6MWD) over the period of a three-month rehabilitation program. When significant, a post hoc Bonferroni adjustment investigated the pairwise comparison of the different measurements. The Friedman test was used as a non-parametric alternative to assess differences in ordinal variables over time. A paired-samples t-test was used to determine whether there was a statistically significant difference between CPET at the start and at the end of the rehabilitation program. Continuous variables were compared between groups with the Student’s t-test or Mann-Whitney U test as appropriate. A chi-square test was used to compare categorical variables between groups. A three-way-mixed ANOVA was performed to determine the influence of categorical variables on the progression in 6MWD over the period of a three-month rehabilitation program between groups. Patients unable to complete the CR program, due to health-related problems, lack of motivation, work commitment, or family commitment, failed to complete all five 6MWTs and were therefore excluded from analysis. Only patients who completed the five 6MWT measurements were included in the statistical analysis. The statistical significance was always set at a 2-tailed probability level of <0.05. Statistics were performed using SPSS version 24 (IBM, Chicago, IL, USA).

## 3. Results

### 3.1. Demographics and Baseline Population

**Figure A1:**
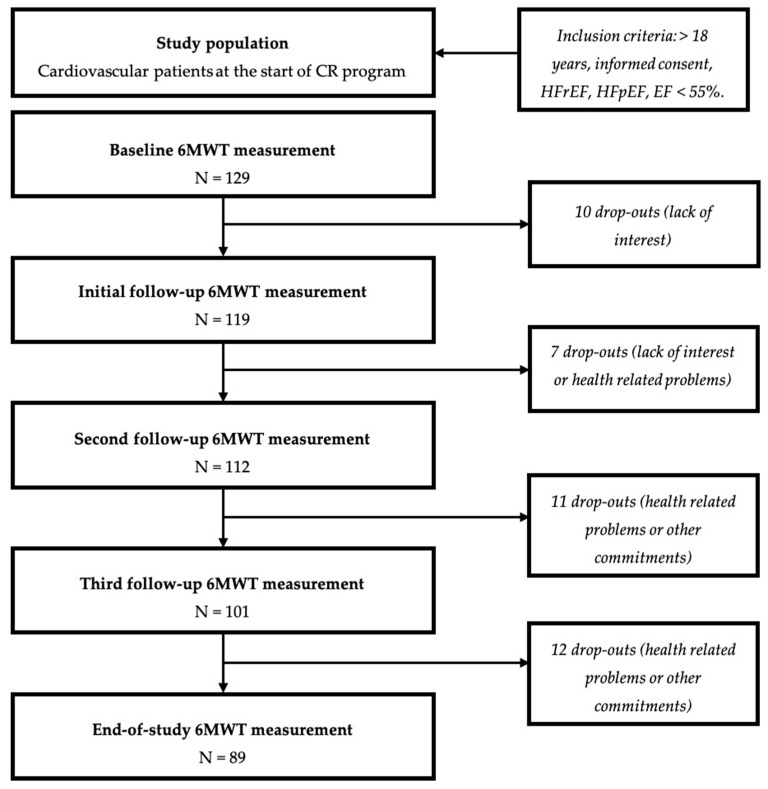
Study population flow chart.

One-hundred and twenty-nine patients, enrolled in the multidisciplinary CR program, consented to participate in the study. Sixty-nine percent of the patients (*n* = 89) completed all five 6MWTs. Patients unable to complete the CR program, due to health-related problems, lack of motivation, work commitment, or family commitment, failed to complete all five 6MWTs and were therefore excluded from analysis when investigating progression throughout the complete CR (Figure A1).

Clinical and baseline characteristics of the patients are summarized in Table 1. Mean age was 63 years and 71.3% of patients were male. Twenty-two percent were actively smoking during the rehabilitation program. Baseline median EF was 45% for the total population and 42% suffered prior myocardial infarction. Twenty-eight percent of patients were classified as NYHA I, 46.5% as NYHA II, and 25.6% as NYHA III. Fifty-one percent of subjects were on ACE-I therapy, 75.2% were also on beta-blocker therapy, 51.9% were taking diuretics, and 72% were on statins therapy. The mean baseline peak VO_2_ was 16.21 mL kg-1min-1, while 6MWD at baseline was 468 m. Reasons of referral were post myocardial infarction (24.8%), HF (29.5%), coronary artery bypass grafting (CABG) (13.2%), percutaneous coronary intervention (11.6%), other (20.9% (Table A1)). There was no statistical significant difference in baseline physical performance, i.e., peak VO_2_ and 6MWD, between referral groups.

One-hundred-and-two patients were referred to CR after hospitalization (79%). The time-delay after hospitalization until the start of the program was on average 21 (18) days.

Table A2 shows the baseline characteristics for the patients who completed the five 6MWTs (*n* = 89) and the patients who failed to finish the study protocol (*n* = 40). Forty-three percent of patients who dropped-out early were referred to CR for HF, compared to 24% in the group who completed the study protocol. The distance walked during the baseline 6MWT was significantly lower for the patients who did not complete the study protocol (484 vs. 431 m). Moreover, peak VO_2_ values and peak power were significantly lower (17.00 vs. 14.06 mL/kg/min and 114 vs. 96 watts) for the drop-out group. Overall compliance rate to the exercise program was 91.9%, with respect to a total of 45 sessions that needed to be followed for the 89 patients who completed the five 6MWTs. While exercise adherence on a three-weekly basis (with respect to 3 sessions per week, 9 in total), in between the consecutive 6MWTs, changed respectively from 86.3% to 86.6%, to 82.8% and to 83.5%.

### 3.2. Measuring Exercise Capacity and HRQoL: CPET Data and 6MWT

For the 89 patients who completed the whole study protocol, mean peak VO_2_ increased significantly with 3.93 (95% CI, 2.60 to 5.32) ml kg^−1^min^−1^ between baseline 16.18 (5.03) mL kg^−1^min^−1^ and end-of-study 20.14 (5.24) mL kg^−1^min^−1^ (t(38) = 5.86, *p* ≤ 0.001, d = 0.94). An improvement in HRQoL was seen after completing CR. More specifically, a significant difference was seen for both MLHFQ and SF-36 questionnaires when comparing baseline with end-of-study measurements. A significant decrease of 14.13 (95% CI, 9.54 to 18.72, t(75) = 6.13, *p* ≤ 0.001, d = 0.70) in total MLHFQ score and a significant increase for almost all SF-36 subclasses, except for the general health subclass, was noted (Table 2). No significant change in blood pressure between baseline and end-of-study measurements was seen (127/75 (19/11) vs. 128/74 (17/12)).

The short-term change in exercise capacity at different time intervals throughout the CR program was studied by means of the 6MWT. A total of 89 patients completed the whole study protocol and performed all five 6MWTs (Table 3).

**Figure 1:**
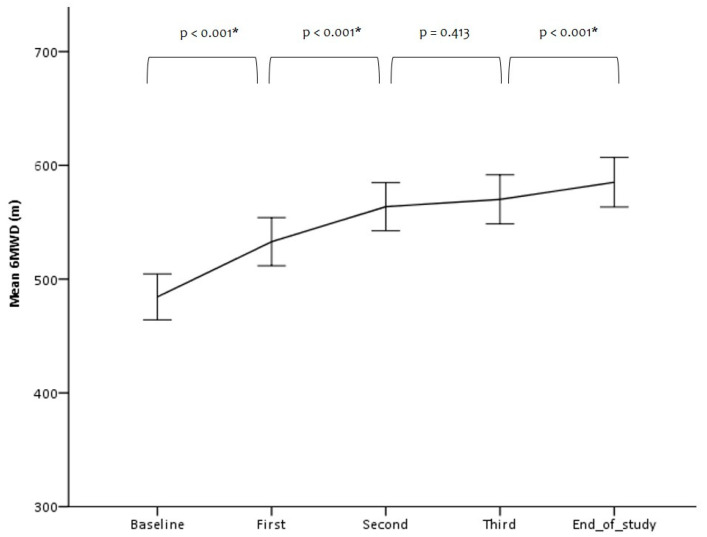
Means and standard deviations of six-minute walking distance (6MWD) for each measurement session throughout the rehabilitation program. * *p* < 0.05.

Based on the one-way repeated measures ANOVA, mean distance differed significantly across consecutive time points during rehabilitation, F (2.357, 207.421) = 164.828, *p* ≤ 0.001, partial η^2^ = 0.652. Post hoc analysis revealed significant differences in 6MWD for each pair of consecutive time points, except for the mean distance between the second and third follow-up measurement. Respectively, an increase of 48.5 (95% CI, 36.0 to 60.9, *p* ≤ 0.001,) m was seen between baseline and first follow-up, 30.8 (95% CI, 20.5 to 41.1, *p* ≤ 0.001) m from first to second follow-up, 6.4 (95% CI, −2.5 to 15.3, *p* = 0.413) m from second to third follow-up and 15 (95% CI, 7.3 to 22.8, *p* ≤ 0.001,) m from third follow-up to end-of-study measurement. The distance tended to increase throughout the exercise program (Figure 1). However, towards the end of the study, the slope decreases.

Fluctuations in HR measured before and after the 6MWT (HR_before_ and HR_after_) and changes in NYHA classification during rehabilitation can provide additional information on exercise capacity in patients diagnosed with cardiovascular disease [30,31]. HR_before_ altered across the consecutive time points during rehabilitation, F (4, 19945) = 2.950, *p* ≤ 0.021, partial η^2^ = 0.038. A significant decrease of 4 bpm from baseline to end-of-study was observed (95% CI, 0 to 8, *p* = 0.024). Although no significant difference could be determined between pairs of mean HR_after_, one-way repeated measures ANOVA showed a significant increase in HR_after_ during the exercise program (F (4, 292) = 3.747, *p* ≤ 0.001, partial η^2^ = 0.049). Friedman testing showed a significant decrease in NYHA classification at the different time points, χ^2^(4) = 36.314, *p* ≤ 0.001 (Baseline: 1.93 (0.70), first: 1.74 (0.70), Second: 1.64 (0.66), Third: 1.56 (0.68), End-of-study: 1.48 (0.67)).

### 3.3. Different Levels of Response to CR

**Figure 2:**
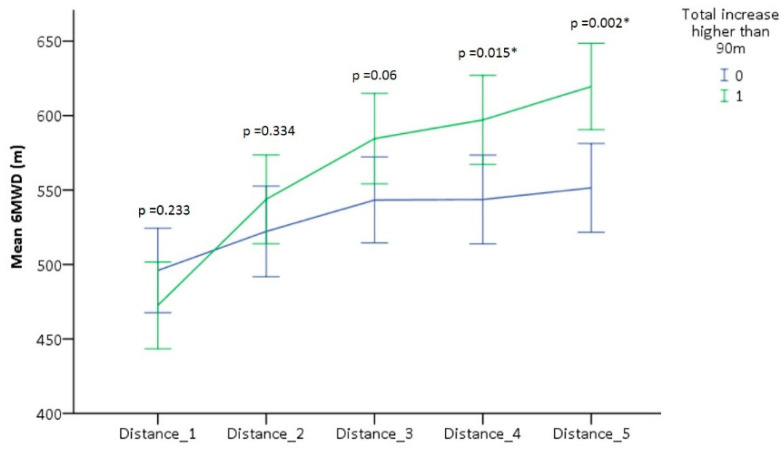
Means and standard deviations of six-minute walking distance (6MWD) walked during rehabilitation for subgroups based on the increase in distance throughout rehabilitation. * *p* < 0.05.

Next, to determine whether the decrease in progression during the second half of the CR program was characteristic for all patients, the increase in 6MWD was investigated on a patient level. Each individual patient was characterized by a unique progression pattern throughout the CR program, representing the complexity of this study population. Therefore, a median split for the increase in distance walked throughout the rehabilitation program, was performed. Two groups with an equal number of patients were created based on an improvement in 6MWD of more or less than 90 m (median value). Two progression patterns emerged (Figure 2).

The group who increased less than 90 m throughout rehabilitation almost plateaued in 6MWD during the second half of rehabilitation, while the other group showed a near-linear progression rate until the end. The less than 90 m group increased 26 (28) m between baseline and first follow-up, 21 (25) m between first and second follow-up, 0 (31) m between second and third follow-up and 8 (22) m between third follow-up and end-of-study. The more than 90 m group increased 71 (39) m between baseline and first follow-up, 41 (39) m between first and second follow-up, 13 (26) m between second and third follow-up and 23 (27) m between third follow-up and end-of-study An increase of 35 ± 34 m was seen for the linear progression group between the second follow-up measurement and end-of-study. This increase differed significantly from the 8 ± 25 m change of the less than 90 meters’ group (95% CI, 14.3 to 39.4, *p* ≤ 0.001). There were no statistically significant differences between both groups with respect to the demographics and baseline characteristics, except for diabetes (11 (24.4%) in less than 90 m group vs. 1 (2.3%) in more than 90 m group). Moreover, no difference in peak VO_2_ and respiratory exchange ratio (RER) were observed (17.2 (5.3) mL/kg/min with a RER of 1.1 (0.1) for the less than 90 m group vs. 16.8 (4.9) mL/kg/min with a RER of 1.1 (0.1) for the more than 90 m group). The reasons of referral were equally divided between both groups. No significant difference in compliance rate was observed between both groups. However, a significant difference between both groups was seen in distance increased between baseline and the initial follow-up measurement. Patients who increased more than 90 m throughout rehabilitation, increased more than 70 m from baseline to first follow-up. The patients who improved less than 90 m during CR, increased less than 30 m between baseline and first follow-up measurement (mean difference of 45.02 m, 95% CI, 30.64 to 59.42, *p* ≤ 0.001).

## 4. Discussion

The current study adds to the knowledge on the short-term progression in exercise capacity of patients following a multidisciplinary CR program. An increase in 6MWD between consecutive 6MWTs throughout the CR was seen, which confirmed the beneficial effects of exercise training.

Our study population increased on average 101 m in 6MWD when comparing baseline and end-of-study measurements. This is a large increase when compared to the outcome of two meta-analysis investigating the effects of CR in a HF population, where an increase of 41 m and 50 m, respectively, was noted [32,33]. On the other hand, a larger improvement was seen in a study investigating the increase in 6MWD after CR in a population with a recent history of acute coronary syndrome [34]. Although only a limited amount of data is available in current literature, making it difficult to define a clinically significant increase in 6MWD, it is clear that it differs among and between patient populations. Moreover, it is dependent on patient characteristics, emphasizing the need to interpret improvement in 6MWD on an individual patient level. Additionally, there was an increase of 4 bpm in HR measured immediately after the 6MWT when comparing baseline with end-of-study measurements. These HR measurements are collected at specific and discrete moments in time. The absence of continuous HR monitoring makes it difficult to determine the cause of this increase. It is possible that patients are more motivated and try harder, causing their HR to increase. However, together with the decrease in the resting HR and the increase in peak VO_2_, it can be stated that the difference in HR is most likely caused by an improvement in fitness level [35]. An improvement in HRQoL was seen after completing CR, except for the general health status subclass. These results indicate that patients do not evaluate their personal health differently at the end of CR when compared to the start of CR. However, they do evaluate their personal health differently when compared to healthy subjects. In other words, they do not consider themselves as healthy individuals and feel compromised by the disease. Although both their mental and physical status improve, CR patients still feel different when compared to healthy subjects after following a CR program. There is limited evidence available on studying this subtopic of the SF-36 questionnaire in this specific patient population. In most research, focus is only put on the average increase in mental and physical health with respect to SF-36 results, no subtopics are discussed.

During the second half of rehabilitation the increase in 6MWD levelled-off. The 6MWT is an outcome measure representative for exercise tolerance, limited by several cardiovascular related and non-cardiovascular related factors, which could all influence the decrease in progression. Firstly, an increase in EF can be present throughout the CR program [36]. It is possible that similar to the 6MWD, a decrease in EF improvement is seen, explaining the stagnation of progression. However, Perreti et al. investigated the relationship between functional improvement (evaluated by a 6MWT) and the improvement in EF after CR in a population with a recent history of acute coronary syndrome. Although patients with a lower EF showed a larger increase in distance walked after CR compared to patients with a higher EF (168 m vs. 194 m), no significant correlation was found [34]. Another possible explanation is that the 6MWT is a submaximal exercise test limited by the patients’ maximal walking speed, thereby limiting the distance they can cover in six minutes [37]. Moreover, osteoarticular pathology or skeletal muscle malfunctioning are determinants of exercise capacity and could influence the rate of improvement. Non-cardiovascular related factors are equally important to consider when studying the decrease in progression. Patient motivation, effort, and willingness to perform the exercise could influence the outcome of a 6MWT [38]. In our study, although not statistically significant, a decreasing trend in compliance rate was seen throughout the program, which could potentially explain the reduced improvement. There are several other known non-cardiovascular factors that can potentially influence the response of patients to CR, which can however often be related to failure to participate [39]. However, in our heterogeneous study population it is difficult to define these (non-) cardiovascular factors that determine the decrease in progression during the second half of CR.

Next, the differences between the patients who completed all five 6MWTs and the patients who failed to finish the study protocol were investigated. Notably, almost half of the patients who failed to complete the study protocol, were patients who were referred to CR for HF. The high number of HF patients in the drop-out group can be a possible explanation for the differences observed during baseline CPET testing. Patients from the drop-out group are characterized by a lower exercise capacity. Ritchey et al. reported similar results showing low participation rates for HF patients [40]. These high drop-out rates for HF referral patients can be explained by the fact that these patients tend to be older and sicker, making them less motivated to finish the rehabilitation program. An additional explanation can be found in the fact that non-procedure related secondary events as a reason for referral, are often characterized by low participation rates. The secondary events are typical the motivation behind referring HF patients to CR [40].

To investigate whether the decrease in progression was characteristic for all patients, the increase in 6MWD was investigated on a patient level. A unique progression pattern for each individual patient was seen throughout the CR program, representing the complexity of this study population. Therefore, patients were divided into two groups by means of a median split based on the increase in distance walked throughout the rehabilitation program. Two different progression patterns emerged. The group who increased more than 90 m showed a near-linear improvement, while the other group’s progression levelled off during the second half of the rehabilitation program. Limited improvement can be associated with female gender and body mass index [41]. However, there were no differences in baseline characteristics between both groups except for the prevalence of diabetes. Previous research groups showed that diabetes patients benefitted less from CR when compared to patients who did not have diabetes, which can be explained by a dysregulation of cardiovascular and metabolic functions [42,43,44]. The lack of improvement in exercise capacity can potentially be explained by genetic factors but also by a decline in motivation or cardiac related barriers causing a decrease in adherence. Therefore, a personalized approach consisting of exercise training programs adjusted to individual needs is recommended in these patients [45]. A higher prevalence of diabetes in the group who improved less than 90 m could (at least partially) explain the difference in progression between both groups. Although it is difficult to determine at baseline which patients will benefit more, a distinction can be made early on in CR. An important difference between the two progression pattern groups is the improvement in 6MWD obtained during the initial three weeks of CR. Patients increasing on average more than 60 m between the initial two measurements, will improve more throughout CR, while patients improving less than 30 m will progress less. These results showcase the potential importance of short-term follow-up, which could allow a fast optimization of exercise management, possibly improving outcome for specific patient subgroups. Future studies should investigate whether similar progression patterns emerge in both center-based (including with larger patient groups) and in home-based CR programs and whether this short-term information on progression can be used to optimize outcomes by improving exercise capacity and motivation.

The results from this observational study can be used as a framework for future studies to compare the short-term progression in functional capacity between center-based and home-based rehabilitation studies.

The authors of this study believe that continuous monitoring of various physiological parameters throughout a 6MWT at the start of CR could provide even more information needed to fine-tune exercise management in a home-based rehabilitation program. Previous research demonstrated that additional parameters optimize the interpretation of the results of a 6MWT. Heart rate recovery, measured immediately after exercise, can function as a powerful prognosticator in cardiovascular disease. Heart rate recovery was shown to be a significant predictor of adverse events and strong predictor of survival [46]. Furthermore, information extracted from accelerometer data can be used to reliable evaluate exercise performance, allowing routine assessment in an in-home setting [27]. Step frequency and activity counts, both parameters extracted from accelerometer data have been proven to be correlated to the 6MWD. Moreover, the activity counts can be used to assess walking speed in a HF population [27]. Our research group showed that wearable sensor technology can be used to characterize response to CR by measuring heart rate parameters and digital biomarkers, derived from continuous measurements. These digital biomarkers allow for in-depth insights into the cardiac response of patients during a standardized activity [47]. Therefore, wearable and mobile technologies, capable of continuously monitoring patients following CR during exercise, could give more insight in the actual progression on patient level throughout rehabilitation. These technologies are not able to provide the same clinical insights as compared to the CPET measurements. The ability of deriving respiratory variables, i.e., oxygen consumption, that would enable insights into the pathogenic mechanisms that lead to exercise limitations (dyspnea and fatigue sensation) are restricted. However, they can expand the capacity of the 6MWT to assess and monitor patients at home by monitoring activity levels, distance walked, and HR measured during exercise, e.g., device-generated data showed a similar effect on HF related events compared to 6MWD alone [48].

This study should be interpreted in the light of some limitations. First, the learning effect of the 6MWT should be taken into account when interpreting the change in distance between baseline and first follow-up measurement [26]. Although a larger increase was seen between the initial two measurements, the increase persists during the following 6MWTs ensuring an improvement in functional capacity. Other limitations to the 6MWT include the difficulty to determine the causes or mechanisms of exercise limitations, compared to the CPET. Moreover, the performance of the patient is motivation dependent [25]. Secondly, some limitations need to be considered on the exercise modalities used to assess functional capacity. During the 6MWT patients can potentially reach an exercise capacity level that makes the 6MWT unsuitable to follow up progression as maximal walking speed can limit their performance [37,49]. The low number of patients who performed a CPET halfway into the rehabilitation program is an important limitation, as it would be of interest to compare these data with the outcome of the 6MWTs. The low number of patients, missing information on marital and educational status, the heterogeneous character of the population, the lack of information on biochemical parameters, and the imbalance between male and female patients are all limitations of the study that need to be taken into account when interpreting the results. In addition, the absolute number of patients screened for study participation and their characteristics have not been described. Another limitation to the study is that changes in medication during the study period were not accounted for. However, it should be mentioned that the 6MWT can be used for the assessment of changes in symptoms, but there are still some hinderances concerning its full scope in terms of assessment of pharmacological interventions [50]. Furthermore, the lack of comparison with a group undergoing home-based CR makes it difficult to implement these results in home-based rehabilitation. However, these interpretations are exploratory and future studies with a matched patient group undergoing home-based CR should be considered. The differences between the patient groups who completed the study protocol and those who did not should be taken into account as almost a third of the patients initially enrolled in the study did not complete the protocol. Especially when considering the present data as a framework for future studies, it is important to emphasize that these results only apply to a subset of patients which were less likely to be referred due to HF and which presented a higher functional capacity at baseline. The results of this study should be investigated in a larger patient group.

## 5. Conclusions

The current study investigated the effect of CR on the progression of exercise capacity in patients diagnosed with cardiovascular disease within short-time spans. The main findings of the study were: (1) After six weeks of initial progression in exercise capacity, a flattening of improvement is reached; (2) Two different patterns of progression emerge when analyzing the data on a more personal level. The group who progressed most, showed a near linear improvement in exercise capacity throughout CR. The improvement of patients who responded less to exercise training, leveled of half-way into rehabilitation. Future studies could use this information to optimize short-term guidance and treatment strategies, namely in home-based CR programs.

## Figures and Tables

**Table 1:** Baseline characteristics.

Variable	Total Population (*n* = 129)
**Demographics**	
Male	92 (71.3%)
Age, years >65 years	63 (11) 55 (42%)
Height, m	1.72 (1.70–1.74)
BMI, kg/m^2^	27.2 (5.0)
Active smoker	28 (22%)
Past smoker	47 (37%)
**LV ejection fraction baseline, %**	45 (42–47)
**LV ejection fraction final, %**	50 (47–53)
**CRT, %**	26 (20%)
CRT-D	15 (58%)
CRT-P	11 (42%)
**Reason of referral**	
MI, % HF, %	32 (24.8%)
	38 (29.5%)
CABG, %	17 (13.2%)
PCI, %	15 (11.6%)
Other, %	27 (20.9%)
**Comorbidities**	
Atrial fibrillation	33 (25.6%)
Hypertension	60 (46.5%)
Dyslipidemia	53 (41.1%)
Diabetes	21 (16.3%)
**NYHA class**	
Class I	36 (27.9%)
Class II	60 (46.5%)
Class III	33 (25.6%)
**Medications**	
ACE inhibitor	67 (51.9%)
Beta-blocker	97 (75.2%)
Diuretics	66 (51.2%)
Statins	93 (72%)
Calcium-channel blockers	10 (8%)
Anticoagulants	42(32%)
**Baseline CPET (*n* = 101)**	
Peak VO_2_, mL/kg/min	16.21 (5.33)
Percent predicted peak VO_2_, %	58 (19)
Peak power, watts	109 (102–115)
Respiratory exchange ratio	1.09 (1.08)
**Baseline 6MWT**	
Distance, m	468 (104)
Rest HR, bpm	68 (65–70)
Rest blood pressure, mmHg	127/75 (19/11)

LV, left ventricular; CRT, cardiac resynchronization therapy; CRT-P, cardiac resynchronization therapy pacemaker; CRT-D, cardiac resynchronization therapy defibrillator. NYHA, New York Heart Association; ACE, angiotensin converting enzyme; CPET, cardiopulmonary exercise testing; 6MWT, six-minute-walking test; HR, heart rate. The information (except for the information obtained during the 6MWT) was collected from the electronic medical record. Information for only 101 CPET measurements were available.

**Table 2:** Output paired sample t-test analyzing the difference in Short Form-36 (SF-36) scores between baseline and end-of-study.

SF-36 Scores (Baseline—End-of-Study)	Mean (SD)	95% CI	t-Value	*p*-Value	Effect Size
Physical Functioning	−14.09 (19.22)	−18.58 to −9.61	−6.26	≤0.001	−0.73
Role Physical	−27.41 (46.99)	−38.22 to −16.60	−5.05	≤0.001	−0.58
Role Emotional	−14.02 (42.71)	−25.35 to −2.69	−2.48	0.016	−0.33
Vitality	−13.22 (17.85)	−17.30 to −9.13	−6.45	≤0.001	−0.74
Mental Health	−9.20 (15.44)	−12.73 to −5.67	−5.20	≤0.001	−0.60
Social Functioning	−20.31 (21.67)	−26.61 to −14.02	−6.50	≤0.001	−0.94
Bodily Pain	−10.44 (24.47)	−17.39 to −3.49	−3.02	0.004	−0.43
General Health	−2.61 (17.72)	−8.89 to 3.67	−0.85	0.404	−0.15

**Table 3:** Output 6MWT at five different time points during rehabilitation.

	Baseline	1st Follow-Up	2nd Follow-Up	3rd Follow-Up	End-of-Study
Distance, m	484 (96)	533 (100)	564 (100)	570 (103)	585 (104)
HR start 6MWT	66 (57–80)	67 (57–73)	65 (58–72)	64 (55–70)	62 (55–72)
HR end 6MWT	85 (73–94)	86 (77–96)	85 (77–103)	89 (80–99)	89 (79–104)

6MWT, six-minute-walking test. Values are expressed as mean (standard deviation) or as median (interquartile range).

## Appendix A

**Table A1:** Specifying “Other” reasons for referral.

Reason for Referral, Others	(*n* = 129)
Non-ischemic cardiomyopathy	9 (7%)
Low fitness level	7 (5%)
Valve disease	5 (4%)
Following CRT implantation	6 (4%)

**Table A2:** Baseline characteristics for patients who completed all five 6MWTs (Complete) and patients who failed to finish the study protocol (Non-complete).

Variable	Complete (*n* = 89)	Non-Complete (*n* = 40)	*p*-Value
**Demographics**			
Male	65 (73%)	27 (68%)	0.534
Age, yrs >65 yrs	63 (10) 37 (42%)	62 (14) 18 (45%)	0.398
Height, m	1.72 (1.70–1.74)	1.71 (1.67–1.74)	0.498
BMI, kg/m^2^	26.7 (4.1)	28.4 (6.3)	0.072
Active smoker	18 (20%)	10 (25%)	0.495
Past smoker	34 (38%)	13 (33%)	0.692
**LV ejection fraction baseline, %**	45 (43–49)	42 (38–46)	0.123
**LV ejection fraction final, %**	50 (47–53)	NA	
**CRT, %**	17 (19%)	9 (23%)	0.627
CRT-D	10 (59%)	5 (50%)	
CRT-P	7 (41%)	4 (50%)	
**Reason of referral**			
MI, % HF, %	23 (26%)	9 (23%)	0.826
	21 (24%)	17 (43%)	0.037 *
CABG, %	14 (16%)	3 (8%)	0.266
PCI, %	11 (12%)	4 (10%)	0.776
Other, %	20 (22%)	7 (16%)	
**Comorbidities**			
Atrial fibrillation	22 (25%)	11 (28%)	0.828
Hypertension	38 (43%)	22 (55%)	0.084
Dyslipidemia	39 (44%)	14 (35%)	0.440
Diabetes	12 (14%)	9 (23%)	0.208
**NYHA class**			0.222
Class I	26 (29%)	10 (25%)	
Class II	44 (50%)	16 (40%)	
Class III	19 (21%)	14 (35%)	
**Medications**			
ACE inhibitor	50 (56.2%)	17 (43%)	0.184
Beta-blocker	63 (71%)	34 (85%)	0.122
Diuretics	39 (43%)	27 (68%)	0.014 *
Statins	64 (71%)	29 (73%)	0.999
Calcium-channel blockers	9 (10%)	1 (3%)	0.172
Anticoagulants	28 (32%)	14 (35%)	0.690
**Baseline CPET (*n* = 101)**			
Peak VO_2_, mL/kg/min	17.00 (5.09)	14.06 (5.46)	0.013 *
Percent predicted peak VO_2_, %	60 (20)	52 (18)	0.373
Peak power, watts	114 (106–122)	96 (84–108)	0.014 *
Respiratory exchange ratio	1.10 (0.10)	1.05 (0.11)	0.061
**Baseline 6MWT**			
Distance, m	484 (96)	431 (110)	0.006 *
Rest HR, bpm	68 (65–71)	67 (63–71)	0.543
Rest blood pressure, mmHg	127/76 (19/10)	124/73 (18/13)	0.320/0.006 *

* *p* < 0.05.
